# Maternal serum zinc level is associated with risk of preeclampsia: A systematic review and meta-analysis

**DOI:** 10.3389/fpubh.2022.968045

**Published:** 2022-08-01

**Authors:** Senjun Jin, Chaozhou Hu, Yanmei Zheng

**Affiliations:** ^1^Emergency and Critical Care Center, Department of Emergency Medicine, Zhejiang Provincial People's Hospital (Affiliated People's Hospital, Hangzhou Medical College), Hangzhou, China; ^2^Center for Reproductive Medicine, Department of Obstetrics, Zhejiang Provincial People's Hospital (Affiliated People's Hospital, Hangzhou Medical College), Hangzhou, China

**Keywords:** zinc, Zn, trace elements, hypertensive disorder complicating pregnancy (HDCP), systematic review, meta-analysis, preeclampsia (PE)

## Abstract

**Background:**

Preeclampsia (PE) is a multi-organ syndrome that onsets in the second half of pregnancy. It is the second leading cause of maternal death globally. The homeostasis of zinc (Zn) levels is important for feto-maternal health.

**Objective:**

We aimed to collect all studies available to synthesize the evidence regarding the association between maternal Zn levels and the risk of preeclampsia.

**Methods:**

A systematic review and meta-analysis was conducted *via* searching seven electronic databases [PubMed, Web of Science, Embase, African Journals Online (AJOL), ClinicalTrial.gov, and two Chinese databases: Wanfang and Chinese National Knowledge Infrastructure, CNKI]. Studies reporting maternal serum Zn levels in pregnant women with or without preeclampsia were included. Eligible studies were assessed through Newcastle-Ottawa Scale (NOS) and the meta-analysis was performed *via* RevMan and Stata. The random-effects method (REM) was used for the meta-analysis with 95% confidence interval (CI). The pooled result was assessed using standard mean difference (SMD). The heterogeneity test was carried out using *I*^2^ statistics, and the publication bias was evaluated using Begg's and Egger's test. Meta-regression and sensitivity analysis was performed *via* Stata software.

**Results:**

A total of 51 studies were included in the final analysis. 6,947 participants from 23 countries were involved in our study. All studies went through the quality assessment. The pooled results showed that maternal serum Zn levels were lower in preeclamptic women than in healthy pregnant women (SMD: −1.00, 95% CI: −1.29, −0.70). Sub-group analysis revealed that geographical, economic context, and disease severity may further influence serum Zn levels and preeclampsia.

**Limitations:**

There are significant between-study heterogeneity and publication bias among included studies.

**Conclusions:**

A lower level of maternal Zn was associated with increased risks of preeclampsia. The associations were not entirely consistent across countries and regions worldwide.

**Systematic review registration:**

https://www.crd.york.ac.uk/prospero/display_record.php?RecordID=337069, Identifier: CRD42022337069

## Introduction

Preeclampsia (PE) is a multi-system disorder that onsets at 20 wks or later in pregnancy. It can affect pregnant women in many ways, such as causing hypertension, proteinuria, liver dysfunction, placental abruption, fetal growth restriction. It is a subtype of the hypertensive disorder spectrum in pregnancy, complicating around 5% of pregnancies worldwide. It remains one of the major causes of maternal, fetal and neonatal mortality, particularly in low-income and middle-income countries (LMICs) (1, 2). Despite many researchers devoted to this field, the etiology of preeclampsia is still largely unknown.

Some scholars proposed that trace elements may play a vital role in developing preeclampsia (3–5). In spite of making up <0.1% in the human body, trace elements have a disproportional function in maintaining health. Overall, evidence suggests that micronutrient imbalances are associated with various disorders (4, 6). This will be more prominent in pregnancy when maternal requirements are usually increased. Optimal supplementation can reduce a range of pregnancies complications, e.g., anemia, gestational diabetes, thyroid disorders, and miscarriage (4).

Zinc (Zn) is one of the essential trace elements. As a micronutrient, it functions as a cofactor for up to 10% of proteins in living organisms, playing a vital role in a range of biological processes in the human body (7). Zn is involved in a range of signaling pathways, e.g., Nuclear Factor Kappa B (NF-κB) signaling, and Toll-like Receptor 4 (TLR4) signaling, carrying considerable clinical implications (8, 9). Diseases such as breast cancer, tuberculosis, and cardiovascular diseases were associated with aberrant levels of serum Zn (10–12). Maternal serum Zn levels are usually measured *via* blood sampling from the maternal antecubital vein, reflecting the maternal homeostasis of Zn (13). During pregnancy, Zn exerts a key role in both maternal physiological adaptations and fetal development. The demand for Zn in fetal growth and placental function increases during the third trimester, and may lead to a lower level of Zn in maternal serum compared to healthy non-pregnant women (14). A significant lower levels of Zn may cause a series of dysfunctions in the biological process and higher risks of developing feto-maternal complications, such as gestational diabetes, preterm pre-labor rupture of membrane (PPROM), preterm birth, and low birth weight. The relationship between maternal serum Zn and preeclampsia were studied as micronutrients imbalance is believed a contributing factor of preeclampsia (3, 15, 16).

In recent years, many studies have explored the association between maternal serum Zn levels and preeclampsia, but the results were inconsistent (17–20). There are geographical, economic and ethnic differences that may explain such disagreement (21–25). In this study, we conducted a systematic review and meta-analysis, including all studies covering the maternal serum Zn levels in preeclamptic and healthy pregnant women (1) to confirm that maternal serum Zn levels were correlated with their preeclamptic risks during pregnancy; (2) to analyze any clues of how geographical locations, economic and ethnic context affect maternal Zn status.

## Methods

### Protocol and registration

This study followed the Preferred Reporting Item for Systematic Reviews and Meta-analysis (PRISMA) Statement. We registered at the National Institution for Health Research with the registration identifier: CRD42022337069, https://www.crd.york.ac.uk/prospero/display_record.php?RecordID=337069

### Search strategy

We searched seven electronic databases [PubMed, Web of Science, Embase, African Journals Online (AJOL), ClinicalTrial.gov, and two Chinese databases: Wanfang and Chinese National Knowledge Infrastructure (CNKI)] from the inception of the databases to May 31st 2022. Two independent reviewers (YM-Z and SJ-J) used a combination of Medical Subject Headings (MeSH) terms and free text words such as “preeclampsia or pre-eclampsia,” “zinc or Zn.” The Chinese databases were approached with equivalent Chinese medical terms. We have manually checked the references of all the full-text articles we had read to complement our study. There were no other restrictions. The detailed search strategies can be accessed in Supplementary Table 1.

### Eligibility criteria and study selection

Studies were included if they were: (1) Observational studies that report maternal serum Zn levels in preeclamptic and healthy pregnant women; (2) the control should be healthy pregnant women instead of gestational diabetic women or non-pregnant women.

Studies were excluded if they were conference papers, editorials, reviews, systematic reviews, or interventional studies.

Study selection was performed by YM-Z and SJ-J. A third reviewer, CZ-H was to resolve any disagreement between the two in study selection.

### Data extraction and quality assessment

Following data were independently extracted by two investigators (YM-Z & SJ-J): Name of the authors, year of publication, types of study design, country of the study population, the number of subjects in the studies, the mean ± standard deviation (SD) of maternal age and serum Zn level in each study. The third investigator (CZ-H) would be consulted once there was disagreement in data extraction or scoring of the quality of studies.

Case-control and cohort studies were assessed according to The Newcastle-Ottawa Scale (NOS, http://www.ohri.ca/programs/clinical_epidemiology/oxford.asp). A score between seven and nine indicates the high quality of a study. Scores ranging from four to six were considered fair quality. A score of three or less suggests that the study was poorly designed. For cross-sectional studies, we applied an adapted form of NOS for structural assessment. The scale ranged from zero to ten. A score of seven to ten indicates good quality. Four to six were considered fair, while a score of three or less was graded as poor quality (26). Rating studies were accomplished by YM-Z and SJ-J, and discussed with CZ-H once there was disagreement in evaluation.

### Sub-group analysis and meta-regression

Sub-group analysis and meta-regression were conducted to evaluate the influence of geographic location, economic development, and disease severity on maternal Zn status, and their corresponding effect on risks of preeclampsia. We categorized four geographical groups primarily based on continents, but Asia was sub-divided into Asia and the Middle-East as there are huge differences in terms of demographical features between the two groups. The final groups were: Africa (Egypt, Kenya, Nigeria, South Africa, Sudan, Zambia), Asia (Bangladesh, China, India, Indonesia, Pakistan), Middle-East (Iran, Iraq, Jordan, Saudi Arabia, Turkey), and others (Australia, Brazil, Croatia, Italy, New Zealand, Poland, and the UK). From an economic perspective, we form two groups, cited from the World Bank classification (27). Group 1 is Low-income and Lower-middle-income economies (LMICs), including Bangladesh, India, Indonesia, Iran, Kenya, Nigeria, Pakistan, Sudan, and Zambia. The countries that were rated as Upper-middle-income economies and High-income economies were allocated to the second group (HMICs), which included Australia, Brazil, Croatia, China, Egypt, Iraq, Italy, Jordan, New Zealand, Poland, Saudi Arabia, South Africa, Turkey, and the UK. Disease severity was applied to those studies with inherent groups of mild and severe disease. We also inspected whether Zn levels were associated with the measurement methods, study types, or geographical locations *via* meta-regression.

### Statistical analysis

This study used Review Manager 5.4.1 (The Nordic Cochrane Center, Copenhagen, Denmark) and Stata version 16.0 (StataCorp, College Station, TX, USA). The serum Zn levels were pooled by standardized mean difference (SMD) with 95% CI to assess the correlation with preeclampsia. The *I*^2^ was used to test the heterogeneity (*I*^2^ ≥ 50% indicates significant heterogeneity), then visualized *via* the forest plot. The random-effect model (REM) was adopted to calculate the combined results if the heterogeneity is considered significant. A sensitivity analysis was performed with the removal of each study once to assess whether any single study could affect the whole outcome. Publication bias was visualized *via* funnel plot with Begg's test and tested with Egger's linear regression.

## Results

### Study selection

**Figure 1:**
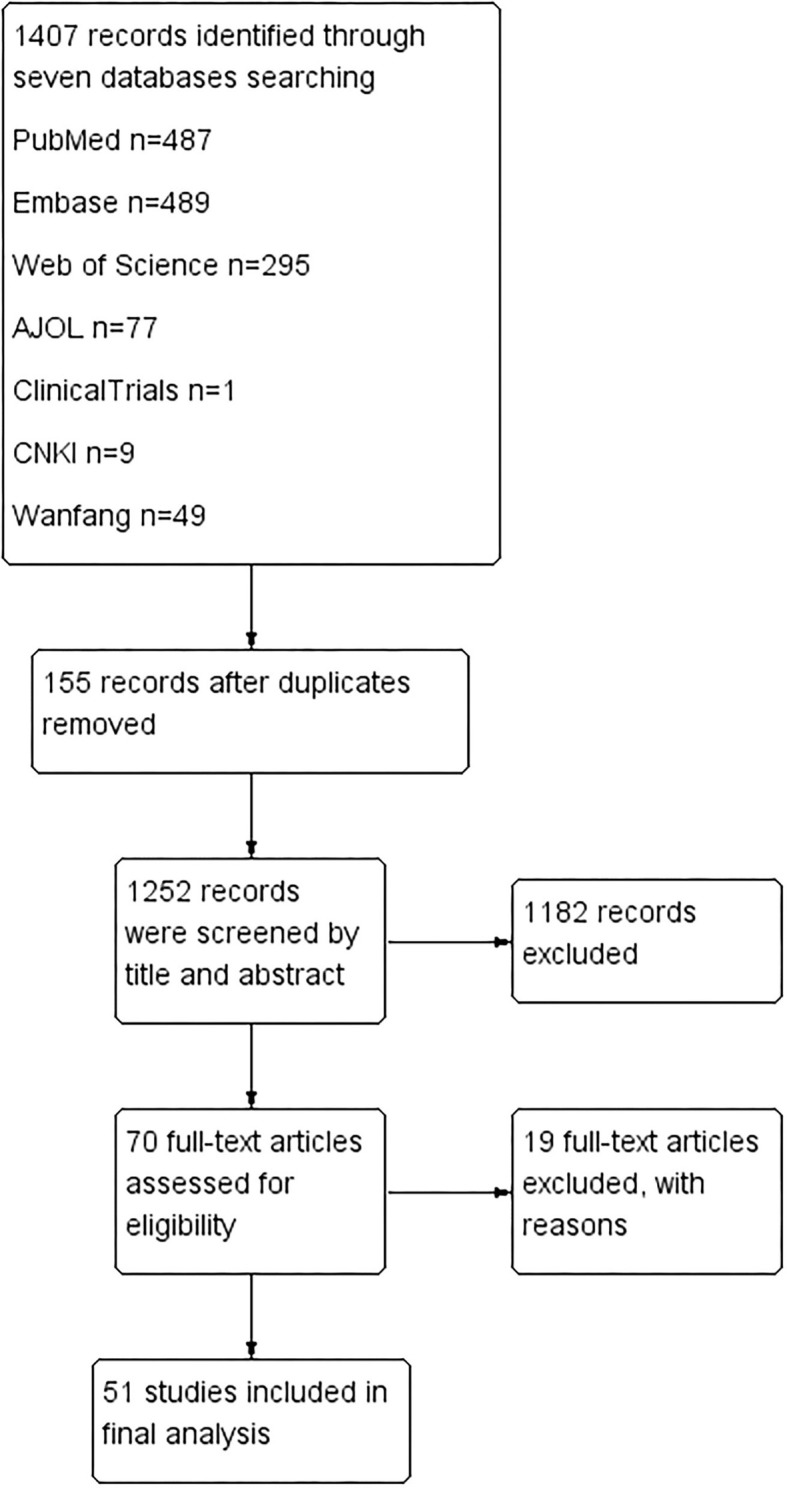
PRISMA flow diagram for study selection process.

One thousand four hundred seven articles were identified after screening seven databases mentioned above. No additional studies were found after checking the references of full-text articles. One hundred fifty-five articles were excluded for duplication. One thousand one hundred eighty-two papers were further ruled out based on title and abstract. After full-text checks, another 19 articles were excluded for the following reasons: (1) Nine were excluded for not answering the research questions; (2) Three were excluded for improper comparison; (3) Two were excluded for improper study types; (4) Five were excluded for inaccessible data. Fifty-one studies were left for quality assessment and data extraction (13, 21–25, 28–72) See Figure 1.

### Basic features of included studies

The 51 studies were conducted across 23 countries over a period of 32 years (1990–2022). Fourty-one studies were case-control, seven were cross-sectional and three were cohort studies. The numbers of preeclamptic women in a single study ranged from 14 to 427 (21, 67) Although not clearly stated in some articles, there was no significant difference in age between the preeclampsia group and the control (21, 30, 37, 39, 40, 46–48, 51, 52, 63, 72). Maternal serum Zn levels were most frequently measured *via* atomic absorption spectrophotometer (AAS) or flame atomic absorption spectrophotometer (FAAS). Other details can be found in Supplementary Table 1.

### Results of systematic review

All studies were classified according to their study designs and further assessed *via* NOS quality assessment tools. Thirty-six (3 cross-sectional, 3 cohort studies and 30 case-control studies) articles were rated as high quality after structured evaluation, while 15 were rated as fair qualified. Detail scores can be accessible in Supplementary Tables 2.1–2.3.

### Results of meta-analysis

**Figure 2:**
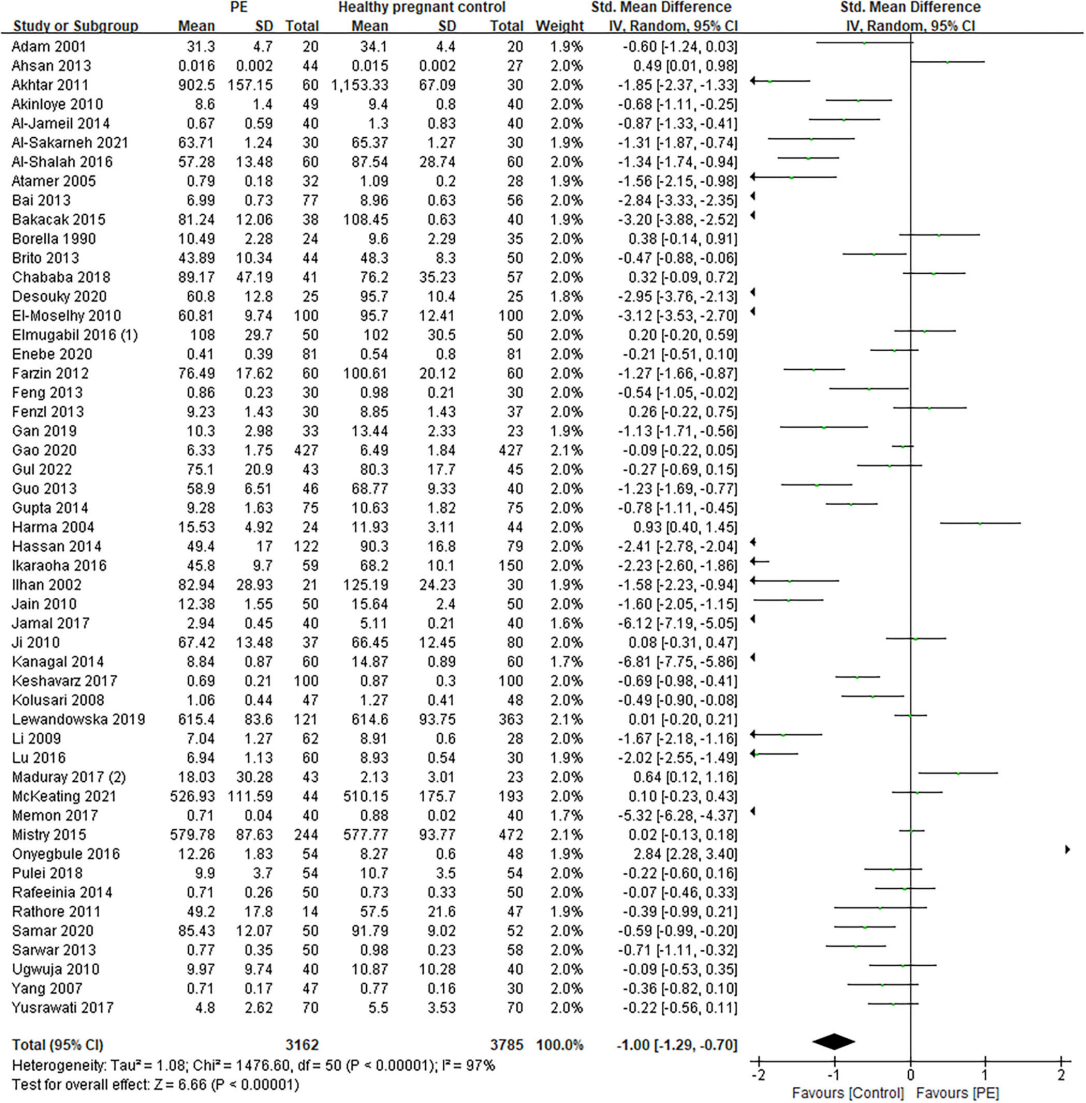
The forest plot for correlation between maternal serum Zn levels in preeclamptic women and healthy pregnant women.

**Figure 3:**
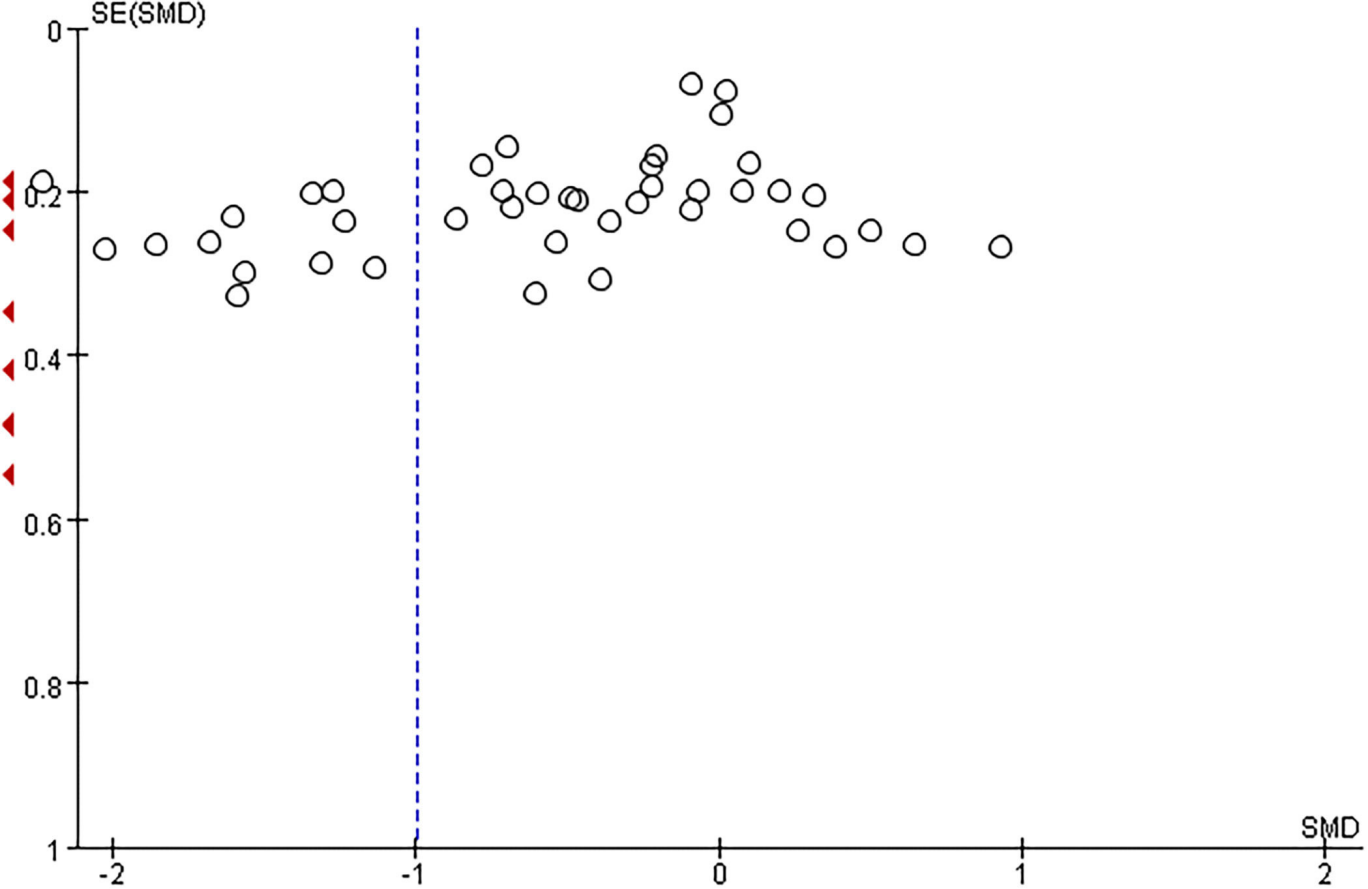
The funnel plot to assess publication bias.

The total number of preeclamptic women involved in this research was 3,162, while the number of participants in the control group was 3,785. The pooled result showed that maternal serum Zn level in preeclamptic women was lower than in healthy control (SMD: −1.00, 95% CI −1.29, −0.70, see Figure 2). The funnel plot can be seen in Figure 3. Begg's test and Egger's test were also performed to assess publication bias, and significant bias was discovered (*z* = 2.88, *p* = 0.004; *t* = −3.89, *p* = 0.000; Supplementary Figure 1). Sensitivity analysis demonstrated that no single study had an overall influence (Supplementary Figure 2; Supplementary Table 3).

### Results of meta-regression

The heterogeneity between studies and across sub-groups was significant. Meta-regression was then performed to explore possible causes. The method of measurement, the geographical locations and the designs of the study types were assessed but the results revealed that the geographical location, study type and different measurement methods were not the causes for heterogeneity (the *P*-value were 0.399 for geographical location, 0.864 for study type, 0.277 for measurement, respectively). Detailed results can be seen in Supplementary Figures 3A–C.

### Results of sub-group analysis

#### Sub-group analysis from a geographic view

Africa has seen the largest number of recent studies. All 12 studies were published since 2010, with 1,465 participants involved. The pooled result was non-significant (SMD −0.65, 95% CI: −1.52, 0.21). Asia is currently home to the largest number of studies and participants (20 studies with 2,665 pregnant women involved), and the result was generally consistent between studies and revealed a negative significance (SMD −1.60, 95% CI −2.13, −1.07). In the Middle-East region, the maternal serum Zn levels were also consistently lower in preeclamptic women compared with normotensive pregnant women, but the difference were not as significant as the population in Asia (SMD −0.93, 95% CI −1.36, −0.49 vs. −1.60, −2.13, −1.07 in Asia). The rest studies were primarily from Europe: one from Italy, one from Croatia, one from Poland, one from Australia, one from Brazil, and one from multi-centers in the UK, Australia and New Zealand. The results were non-significant (SMD 0.02, 95% CI −0.14, 0.18). More detailed results were available in Supplementary Figure 4.

#### Sub-group analysis from an economic view (HIMCs vs. LMICs)

The studies were divided into High-and-Upper-middle-Income countries (HIMCs, 29 studies from 13 countries were included) and Low-and Lower-middle-Income countries (LMICs, 22 studies from 10 countries were included) from an economic perspective. The pooled results of SMD were −0.84 in HMICs vs. −1.23 in LMICs, respectively. The negative association of maternal Zn levels was more notable in developing countries with details accessible in Supplementary Figure 5.

#### Disease severity and Zn levels

Only ten studies have sub-divided the disease of preeclampsia into mild and severe types. The pooled results demonstrated that maternal Zn levels were more negatively associated with disease severity. The SMD was −0.75 (95% CI: −1.36, −0.15) in mild preeclamptic women and −1.32 (95% CI: −2.02, −0.63) in severe form. Other details can be seen in Supplementary Figure 6.

## Discussion

This systematic review and meta-analysis mainly focus on whether there is an association between maternal serum Zn levels and preeclampsia. The overall result demonstrates that a lower level of maternal serum Zn was observed in preeclamptic women than in normotensive pregnant women worldwide. The trend was more prominent in Asian ethnicity, low-income economies and severe patients. This is generally consistent with findings from other reports (17–20).

Zn has many roles in the body, including maintaining the catalytic activity of a range of enzymes, protein synthesis, cell division. It is also involved in the immune system, nerve function, and fertility (4). Its role in the immune system has been well-known for several decades (73). T-lymphocytes activation requires the presence of Zn. Even a mild degree of Zn deficiency can impair macrophage, neutrophil functions, natural killer (NK) activity and complementary response (74). It also helps maintain skin integrity and delay age-related macular degeneration, and vision loss (75, 76). Zn level mainly depends on dietary intake as no specific Zn storage system has been identified (77). The Recommended Dietary Allowance (RDA) of Zn for pregnant women in the US is at least 11 mg/d. Oysters, red meat like beef, poultry, and beans are zinc-rich diet choices. A higher level of zinc-containing supplements may bring more benefits (4). Zn-containing supplements are additional resources for Zn intake. The median level of Zn is 15 mg in prenatal supplements in the US markup-to-datedate evidence suggests zinc sulfate, zinc gluconate or zinc lactate may be beneficial, while zinc acetate should be avoided (4). Despite the maternal serum levels of Zn can be influenced by confounders such as stress and infections, Zn toxicity barely occurs in women with an average daily intake of zinc-containing supplements or food (16). The Tolerable Upper Limit (TUL) is 40 mg daily.

During pregnancy, Zn is essential in embryogenesis and fetal development. Animal studies have shown that Zn deficiency could lead to abnormal placental morphogenesis, which is one of the presumptive etiology of preeclampsia (78, 79). Despite being minimal in serum, the Zn level is consistently lower in preeclamptic women. This indicates that Zn has a role to play in the pathogenesis of this pregnancy-specific disorder. There are studies suggest Zn as an antioxidant trace element, can relieve the oxidative stress in rats (78, 80). Oxidative stress is believed one of the key pathogenesis in the development of preeclampsia. A more recent study has identified that Zn may also participate in ferroptosis, a newly-discovered iron-dependent form of non-apoptotic cell death (81, 82). Furthermore, as there has been no solid evidence to demonstrate a preventive effect of Zn supplements to reduce the risk of preeclampsia, clinical research can be conducted to explore the possibility that Zn takes part in ferroptosis to mediate the development of preeclampsia (83).

Despite four meta-analysis having reached similar conclusions that maternal serum Zn levels were lower in preeclamptic women, there were reasons why we updated the evidence and added more information (17–20). (1) Three meta-analysis were conducted before 2016, but 20 articles we had included were after that (21–24, 32, 39, 41, 42, 46, 51, 54, 57, 60–65, 68, 72). This indicates there are still unclear or unreasonable phenomena and different conclusions may be drawn with more evidence accumulated and careful analysis. (2) The other meta-analysis, conducted last year focused on the African population while we have a global perspective. By comparison, we give more information for researchers who also care about the rest of the world. (3) All the four meta-analysis did not include adequate articles even in their claimed scope. We found more than 30 studies reporting a relationship between maternal Zn levels and preeclampsia by 2016 using almost the same search strategy as Zhu et al. (17, 18, 20). We had involved one more African study compared to Tesfa et al. (61) (4) We have the most complete sub-group analysis. Zhu et al. had sub-group analysis in terms of study design and geographical locations, but they only include a total of 13 studies (20). Ma et al. revealed a sub-group analysis in terms of continent (Asia, Europe, Africa), sample type (plasma, serum), fasting status (yes or no), individual age match or gestational age match) (18). These results of sub-group analysis may be extrapolated cautiously as the number in each group was relatively small. He et al. did not involve sub-group analysis (17). In general, we have synthesized the most up-to-date evidence, applied sub-group analysis to identify more information to encourage further research.

However, our studies have several limitations. First, we claim to have a global view, but there was scanty evidence from Latin America or some non-English speaking European countries. This is due to that we have not searched the non-English databases. However, we have involved all the possible database we can have, including a pure African database and two Chinese databases to complete a global view. The English language accounts for most world's existing research articles, and we did not preclude non-English literature in the major database (i.e., PubMed, WOS, Embase). Therefore, we believe that we are very near to all the related literature available in the world. Second, only six western countries were included in our studies. This is less convincing to draw a global conclusion without obtaining enough evidence from an important part of the world (23, 25, 37, 45, 62). This may also reflect that the micronutrients have not been a focus in developed countries anymore as there is scarce research currently conducted in conventional western countries. Third, the between-study heterogeneity was significant even though we had considered different definitions of preeclampsia, different methods of measurement, participants' fasting status, and various conditions for storage. However, it was also reflected in other similar meta-analysis covering the correlation between trace elements or vitamins and preeclampsia (84–86).

## Conclusion

In summary, we have confirmed that maternal serum Zn levels are negatively associated with preeclamptic risk. This correlation is more prominent in Asian countries and low-income economies and is also inversely related to the severity of preeclampsia. Well-designed large cohort or interventional studies in the future may explore why and how maternal serum Zn levels affect the risk of preeclampsia.

## Data availability statement

The raw data supporting the conclusions of this article will be made available by the authors, without undue reservation.

## Author contributions

SJ and YZ: conceptualization, methodology, software, investigation, resources, project administration, modification, and writing back to reviewers. SJ and CH: validation, formal, analysis, and data curation. SJ: writing original draft preparation. CH: writing review, editing, and supervision. YZ: visualization and funding acquisition. All authors have read and agreed to the published version of the manuscript.

## Funding

This research was co-supported by Zhejiang Provincial Project for Medical and Health Science and Technology, grant number 2019323925 and 2021441040.

## Conflict of interest

The authors declare that the research was conducted in the absence of any commercial or financial relationships that could be construed as a potential conflict of interest.

## Publisher's note

All claims expressed in this article are solely those of the authors and do not necessarily represent those of their affiliated organizations, or those of the publisher, the editors and the reviewers. Any product that may be evaluated in this article, or claim that may be made by its manufacturer, is not guaranteed or endorsed by the publisher.

## References

[1] Chappell LC Cluver CA Kingdom J Tong S. Pre-Eclampsia. Lancet. (2021) 398:341–54. 10.1016/S0140-6736(20)32335-7 34051884

[2] Magee LA Nicolaides KH von Dadelszen P. Preeclampsia. N Engl J Med. (2022) 386:1817–32. 10.1056/NEJMra2109523 35544388

[3] Ren M Zhao J Wang B An H Li Y Jia X . Associations between hair levels of trace elements and the risk of preterm birth among pregnant women: a prospective nested case-control study in beijing birth cohort (Bbc), China. Environ Int. (2022) 158:106965. 10.1016/j.envint.2021.106965 34735958

[4] Adams JB Sorenson JC Pollard EL Kirby JK Audhya T. Evidence-based recommendations for an optimal prenatal supplement for women in the U.S., part two: minerals. Nutrients. (2021) 13:1849–76. 10.3390/nu13061849 34071548 PMC8229801

[5] Gajewska K Blazewicz A Laskowska M Nizinski P Dymara-Konopka W Komsta L. Chemical elements and preeclampsia - an overview of current problems, challenges and significance of recent research. J Trace Elem Med Biol. (2020) 59:126468. 10.1016/j.jtemb.2020.126468 32007824

[6] Bhattacharya PT Misra SR Hussain M. Nutritional aspects of essential trace elements in oral health and disease: an extensive review. Scientifica. (2016) 2016:5464373. 10.1155/2016/5464373 27433374 PMC4940574

[7] Weiss A Murdoch CC Edmonds KA Jordan MR Monteith AJ Perera YR . Zn-regulated gtpase metalloprotein activator 1 modulates vertebrate zinc homeostasis. Cell. (2022) 185:2148–2163. 10.1016/j.cell.2022.04.011 35584702 PMC9189065

[8] Jarosz M Olbert M Wyszogrodzka G Mlyniec K Librowski T. Antioxidant and anti-inflammatory effects of zinc. Zinc-dependent nf-kappab signaling. Inflammopharmacology. (2017) 25:11–24. 10.1007/s10787-017-0309-4 28083748 PMC5306179

[9] Maywald M Wessels I Rink L. Zinc signals and immunity. Int J Mol Sci. (2017) 18:2222–55. 10.3390/ijms18102222 29064429 PMC5666901

[10] Choi S Liu X Pan Z. Zinc deficiency and cellular oxidative stress: prognostic implications in cardiovascular diseases. Acta Pharmacol Sin. (2018) 39:1120–32. 10.1038/aps.2018.25 29926844 PMC6289396

[11] Gengenbacher M Kaufmann SH. Mycobacterium tuberculosis: success through dormancy. FEMS Microbiol Rev. (2012) 36:514–32. 10.1111/j.1574-6976.2012.00331.x 22320122 PMC3319523

[12] Chandler P Kochupurakkal BS Alam S Richardson AL Soybel DI Kelleher SL. Subtype-specific accumulation of intracellular zinc pools is associated with the malignant phenotype in breast cancer. Mol Cancer. (2016) 15:2. 10.1186/s12943-015-0486-y 26728511 PMC4700748

[13] Ahsan T Banu S Nahar Q Ahsan M Khan MN Islam SN. Serum trace elements levels in preeclampsia and eclampsia: correlation with the pregnancy disorder. Biol Trace Elem Res. (2013) 152:327–32. 10.1007/s12011-013-9637-4 23526144

[14] Choi R Sun J Yoo H Kim S Cho YY Kim HJ . A prospective study of serum trace elements in healthy Korean pregnant women. Nutrients. (2016) 8:749–64. 10.3390/nu8110749 27886083 PMC5133131

[15] Kucukaydin Z Kurdoglu M Kurdoglu Z Demir H Yoruk IH. Selected maternal, fetal and placental trace element and heavy metal and maternal vitamin levels in preterm deliveries with or without preterm premature rupture of membranes. J Obstet Gynaecol Res. (2018) 44:880–9. 10.1111/jog.13591 29369445

[16] Wilson RL Grieger JA Bianco-Miotto T Roberts CT. Association between maternal zinc status, dietary zinc intake and pregnancy complications: a systematic review. Nutrients. (2016) 8:641–68. 10.3390/nu8100641 27754451 PMC5084028

[17] He L Lang L Li Y Liu Q Yao Y. Comparison of serum zinc, calcium, and magnesium concentrations in women with pregnancy-induced hypertension and healthy pregnant women: a meta-analysis. Hypert Preg. (2016) 35:202–9. 10.3109/10641955.2015.1137584 26930501

[18] Ma Y Shen XL Zhang DF. The relationship between serum zinc level and preeclampsia: a meta-analysis. Nutrients. (2015) 7:7806–20. 10.3390/nu7095366 26389947 PMC4586561

[19] Tesfa E Nibret E Munshea A. Maternal serum zinc level and pre-eclampsia risk in african women: a systematic review and meta-analysis. Biol Trace Elem Res. (2021) 199:4564–71. 10.1007/s12011-021-02611-7 33527339 PMC8516764

[20] Zhu Q Zhang L Chen X Zhou J Liu J Chen J. Association between zinc level and the risk of preeclampsia: a meta-analysis. Arch Gynecol Obst. (2016) 293:377–82. 10.1007/s00404-015-3883-y 26386964

[21] Gao LY Wang Y Wu WW Feng YL Yang HL Wang SP. Relationship between newborn birth weight and serum zinc of pregnant women with preeclampsia. Chin Remed Clin. (2020) 20:4061–4064. 10.11655/zgywylc2020.24.001

[22] Enebe JT Dim CC Ugwu EO Enebe NO Meka IA Obioha KC . Serum antioxidant micronutrient levels in pre-eclamptic pregnant women in enugu, south-east nigeria: a comparative cross-sectional analytical study. BMC Preg Childbirth. (2020) 20:392. 10.1186/s12884-020-03081-w 32631273 PMC7339396

[23] Lewandowska M Sajdak S Marciniak W Lubiński J. First trimester serum copper or zinc levels, and risk of pregnancy-induced hypertension. Nutrients. (2019) 11:2479–88. 10.3390/nu11102479 31623110 PMC6835641

[24] Gul AZ Atakul N Selek S Atamer Y Sarikaya U Yildiz T . Maternal serum levels of zinc, copper, and thiols in preeclampsia patients: a case-control study. Biol Trace Elem Res. (2022) 200:464–72. 10.1007/s12011-021-02660-y 33704670

[25] Mistry HD Gill CA Kurlak LO Seed PT Hesketh JE Méplan C . Association between maternal micronutrient status, oxidative stress, and common genetic variants in antioxidant enzymes at 15 weeks? gestation in nulliparous women who subsequently develop preeclampsia. Free Rad Biol Med. (2015) 78:147–55. 10.1016/j.freeradbiomed.2014.10.580 25463281 PMC4291148

[26] Modesti PA Reboldi G Cappuccio FP Agyemang C Remuzzi G Rapi S . Panethnic differences in blood pressure in europe: a systematic review and meta-analysis. PLoS ONE. (2016) 11:e0147601. 10.1371/journal.pone.0147601 26808317 PMC4725677

[27] Bank TW. World Bank Country Lending Groups. (2022). Available online at: https://datahelpdesk.worldbank.org/knowledgebase/articles/906519 (accessed March 20, 2022).

[28] Adam B Malatyalioglu E Alvur M Talu C. Magnesium, zinc and iron levels in pre-eclampsia. J Matern Fetal Med. (2001) 10:246–50. 10.1080/jmf.10.4.246.250-14 11531150

[29] Akhtar S Begum S Ferdousi S. Calcium and zinc deficiency in preeclamptic women. J Bangladesh Soc Physiol. (2011) 6:94–9. 10.3329/jbsp.v6i2.9758

[30] Akinloye O Oyewale OJ Oguntibeju OO. Evaluation of trace elements in pregnant women with pre-eclampsia. Afri J Biotechnol. (2010) 9:5196–202.

[31] Al-Jameil N Tabassum H Al-Mayouf H Aljohar HI Alenzi ND Hijazy SM . Analysis of serum trace elements-copper, manganese and zinc in preeclamptic pregnant women by inductively coupled plasma optical emission spectrometry: a prospective case controlled study in Riyadh, Saudi Arabia. Int J Clin Exp Pathol. (2014) 7:1900–10. 24966900 PMC4069901

[32] Al-Sakarneh NA Mashal RH. Evaluation of zinc and homocysteine status in pregnant women and their association with pre-eclampsia in Jordan. Prev Nutr Food Sci. (2021) 26:21–9. 10.3746/pnf.2021.26.1.21 33859956 PMC8027045

[33] Al-Shalah HH Al-Hilli NM Hasan MA. The association of serum iron, zinc, and copper levels with preeclampsia. Med J Babylon. (2015) 12:1027–36.28325649

[34] Atamer Y Koçyigit Y Yokus B Atamer A Erden AC. Lipid peroxidation, antioxidant defense, status of trace metals and leptin levels in preeclampsia. Eur J Obst Gynecol Rep Biol. (2005) 119:60–6. 10.1016/j.ejogrb.2004.06.033 15734086

[35] Bai T. The diagnostic value of trace elements, ldh and ua in hypertensive disorders in pregnancy. Chin J Postgrad Med. (2013) 36:49–52. 10.3760/cma.j.issn.1673-4904.2013.12.019

[36] Bakacak M Kilinç M Serin S Ercan Ö Köstü B Avci F . Changes in copper, zinc, and malondialdehyde levels and superoxide dismutase activities in pre-eclamptic pregnancies. Med Sci Monitor. (2015) 21:2414–20. 10.12659/MSM.895002 26280939 PMC4544335

[37] Borella P Szilagyi A Than G Csaba I Giardino A Facchinetti F. Maternal plasma concentrations of magnesium, calcium, zinc and copper in normal and pathological pregnancies. Sci Total Environ. (1990) 99:67–76. 10.1016/0048-9697(90)90212-D 2270473

[38] Brito JA Marreiro DN Moita Neto JM Costa e Silva DM Almondes KGS Neto JDV . Enzyme activity of superoxide dismutase and zincemia in women with preeclampsia. Nutr Hosp. (2013) 28:486–90. 10.3305/nh.2013.28.2.6179 23822702

[39] Chababa L Mukosha M Sijumbila G Vwalika BV. Relationship between serum zinc levels and preeclampsia at the university teaching hospital lusaka, Zambia. Med J Zambia. (2018) 43:5.

[40] El-Moselhy EA Amin HH El-Aal HMA. Maternal serum calcium and trace elements; copper and zinc among preeclamptic women in cairo, egypt. Egypt J Hosp Med. (2010) 41:11. 10.21608/ejhm.2010.16951

[41] Elmugabil A Hamdan HZ Elsheikh AE Rayis DA Adam I Gasim GI. Serum calcium, magnesium, zinc and copper levels in sudanese women with preeclampsia. PLoS ONE. (2016) 11:e0167495. 10.1371/journal.pone.0167495 27911936 PMC5135106

[42] El Desouky E Raslan OK El Magd IA Al Sheikh W Eltonsy M. Comparative study for serum zinc and copper levels in cases with normal pregnancy versus preeclampsia. Nat Sci. (2020) 18:180–184. 10.7537/marsnsj180120.23

[43] Farzin L Sajadi F. Comparison of serum trace element levels in patients with or without pre-eclampsia. J Res Med Sci. (2012) 17:938–41. 23825993 PMC3698652

[44] Feng JJ Wang YX. The measurement and clinical significance of serum trace elements in early - onset severe preeclampsia. Chin J Birth Health Heredity. (2013) 21:82–4. 10.13404/j.cnki.cjbhh.2013.05.044

[45] Fenzl V Flegar-Meštrić Z Perkov S Andrišić L Tatzber F Žarković N . Trace elements and oxidative stress in hypertensive disorders of pregnancy. Arch Gynecol Obst. (2013) 287:19–24. 10.1007/s00404-012-2502-4 22878906

[46] Gan Y Chen Z Zhang J Liu W Shi Q. Correlation between vitamin C, vitamin E, trace element and preeclampsia during pregnancy. Chin J Clin Obstet Gynecol. (2019) 20:456–7. 10.13390/j.issn.1672-1861.2019.05.029

[47] Guo LL Guo SL Li SX Zhang SY Li HX Niu LH . The study on the relationship between trace elements content in whole blood and hypertensive disorder complicating pregnancy. Chin J Birth Health Heredity. (2013) 4:60–62. 10.13404/j.cnki.cjbhh.2013.11.031

[48] Gupta S Jain NP Avasthi K Wander GS. Plasma and erythrocyte zinc in pre-eclampsia and its correlation with foetal outcome. J Assoc Physicians India. (2014) 62:306–10. 25327032

[49] Harma M Harma M Kocyigit A. Correlation between maternal plasma homocysteine and zinc levels in preeclamptic women. Biol Trace Elem Res. (2005) 104:97–105. 10.1385/BTER:104:2:097 15894810

[50] Hassan EE Elhhatim WS Bakhit SM Shrif NEMA Huneif MA. Assessment of trace elements in sudanese preeclamptic pregnant women. Eur J Biomed Pharm Sci. (2014) 1:8.

[51] Ikaraoha IC Mbadiwe NC Anetor JI Ojareva IA. Serum trace metals in pre-eclamptic nigerians. Asian J Med Sci. (2016) 7:78–83. 10.3126/ajms.v7i3.13027

[52] Ilhan N Ilhan N Simsek M. The changes of trace elements, malondialdehyde levels and superoxide dismutase activities in pregnancy with or without preeclampsia. Clin Biochem. (2002) 35:393–7. 10.1016/S0009-9120(02)00336-3 12270770

[53] Jain S Sharma P Kulshreshtha S Mohan G Singh S. The role of calcium, magnesium, and zinc in pre-eclampsia. Biol Trace Elem Res. (2010) 133:162–70. 10.1007/s12011-009-8423-9 19547932

[54] Jamal B Shaikh F Memon MY. To determine the effects of copper, zinc and magnesium in patients with pre-eclampsia. J Liaquat Univ Med Health Sci. (2017) 16:53–7. 10.22442/jlumhs.171610506

[55] Ji C Bai D Song FX Rong CE Zhang YN Cui YJ. The clinical implication of serum trace elements in patients with hypertensive disorder in pregnancy. Med Inform. (2010) 23:4415–16. 10.3969/j.issn.1006-1959.2010.11.483

[56] Kanagal DV Rajesh A Rao K Shetty H Shetty PK Ullal H. Zinc and copper levels in preeclampsia: a study from coastal South India. Int J Reprod Contracept Obstet Gynecol. (2014) 3:370–3. 10.5455/2320-1770.ijrcog20140617

[57] Keshavarz P Nobakht MGBF Mirhafez SR Nematy M Azimi-Nezhad M Afin SA . Alterations in lipid profile, zinc and copper levels and superoxide dismutase activities in normal pregnancy and preeclampsia. Am J Med Sci. (2017) 353:552–8. 10.1016/j.amjms.2017.03.022 28641718

[58] Kolusari A Kurdoglu M Yildizhan R Adali E Edirne T Cebi A . Catalase activity, serum trace element and heavy metal concentrations, and vitamin A, D and E levels in pre-eclampsia. J Int Med Res. (2008) 36:1335–41. 10.1177/147323000803600622 19094444

[59] Li PZ Li XY. Trace elements in pregnancy-induced hypertension and related diseases research. Guide China Med. (2009) 7:29–31. 10.15912/j.cnki.gocm.2009.10.151

[60] Lu YH Han LJ Zhang L Ni SN Tian QY. Correlation between hypertensive disorder in pregnancy and serum calcium, prostaglandin e, endothelin. Hebei Med J. (2016) 38:1057–9. 10.3969/j.issn.1002-7386.2016.07.032

[61] Maduray K Moodley J Soobramoney C Moodley R Naicker T. Elemental analysis of serum and hair from pre-eclamptic South African women. J Trace Elem Med Biol. (2017) 43:180–6. 10.1016/j.jtemb.2017.03.004 28325649

[62] McKeating DR Fisher JJ MacDonald T Walker S Tong S Bennett WW . Circulating trace elements for the prediction of preeclampsia and small for gestational age babies. Metabol Offic J Metabol Soc. (2021) 17:90. 10.1007/s11306-021-01840-0 34557980

[63] Memon AR Memon FW Akram M Memon PJ Rahman I. Association of serum zinc level with pre eclampsia. J Liaquat Univ Med Health Sci. (2017) 16:58–61. 10.22442/jlumhs.171610507

[64] Onyegbule AO Onah CC Iheukwumere BC Udo JN Atuegbu CC Nosakhare NO. Serum copper and zinc levels in preeclamptic nigerian women. Nig Med J. (2016) 57:182–4. 10.4103/0300-1652.184071 27397960 PMC4924402

[65] Pulei AN Kinuthia J Omondi O. Serum levels of selected micronutrients in primigravida with pre-eclampsia versus their normotensive counterparts. Int J Med Health Sci. (2018) 4:9–17. 10.53555/ephmhs.v4i4.606

[66] Rafeeinia A Tabandeh A Khajeniazi S Marjani AJ. Serum copper, zinc and lipid peroxidation in pregnant women with preeclampsia in gorgan. Open Biochem J. (2014) 8:83–8. 10.2174/1874091X01408010083 25400710 PMC4231371

[67] Rathore S Gupta A Batra HS Rathore R. Comparative study of trace elements and serum ceruloplasmin level in normal and pre-eclamptic pregnancies with their cord blood. Biomed Res India. (2011) 22:207–10. 10.1186/1475-925X-10-23 21457580 PMC3076264

[68] Samar A Wang DL Iskandar X. Correlation between gestational hypertension and serum vitamin D, trace element level. J Xinjiang Med Univ. (2020) 43:597–600. 10.3969/j.issn.1009-5551.2020.05.01529039975

[69] Sarwar MS Ahmed S Ullah MS Kabir H Rahman GK Hasnat A . Comparative study of serum zinc, copper, manganese, and iron in preeclamptic pregnant women. Biol Trace Elem Res. (2013) 154:14–20. 10.1007/s12011-013-9721-9 23749478

[70] Ugwuja EI Ejikeme BN Ugwu NC Obeka NC Akubugwo EI Obidoa O. Comparison of plasma copper, iron and zinc levels in hypertensive and non-hypertensive pregnant women in abakaliki, south eastern nigeria. Pak J Nutr. (2010) 9:1136–40. 10.3923/pjn.2010.1136.1140

[71] Yang LT Gu LP Zhang WY Fan LM. The study on the relationship between hypertensive disorder complicating pregnancy and the serum zinc, copper, iron, maganese. Mat Child Health Care China. (2007) 22:4082–5. 10.3969/j.issn.1001-4411.2007.29.011

[72] Yusrawati Saputra NPK Lipoeto NI Machmud R. Analyses of nutrients and body mass index as risk factor for preeclampsia. J Obstet Gynaecol India. (2017) 67:409–13. 10.1007/s13224-017-0982-7 29162954 PMC5676581

[73] Kay RG Tasman-Jones C. Acute zinc deficency in man during intravenous alimentation. Aust N Z J Surg. (1975) 45:325–30. 10.1111/j.1445-2197.1975.tb05767.x 813623

[74] Kehl-Fie TE Skaar EP. Nutritional immunity beyond iron: a role for manganese and zinc. Curr Opin Chem Biol. (2010) 14:218–24. 10.1016/j.cbpa.2009.11.008 20015678 PMC2847644

[75] Evans JR Lawrenson JG. Antioxidant vitamin and mineral supplements for slowing the progression of age-related macular degeneration. Cochrane Datab Syst Rev. (2017) 7:CD000254. 10.1002/14651858.CD000253.pub4 28756618 PMC6483465

[76] Pfeiffer RL Marc RE Jones BW. Persistent remodeling and neurodegeneration in late-stage retinal degeneration. Prog Retin Eye Res. (2020) 74:100771. 10.1016/j.preteyeres.2019.07.004 31356876 PMC6982593

[77] To PK Do MH Cho JH Jung C. Growth modulatory role of zinc in prostate cancer and application to cancer therapeutics. Int J Mol Sci. (2020) 21:2991–08. 10.3390/ijms21082991 32340289 PMC7216164

[78] Yu Q Sun X Zhao J Zhao L Chen Y Fan L . The effects of zinc deficiency on homeostasis of twelve minerals and trace elements in the serum, feces, urine and liver of rats. Nutr Metab. (2019) 16:73. 10.1186/s12986-019-0395-y 31687040 PMC6820923

[79] Wilson RL Leemaqz SY Goh Z McAninch D Jankovic-Karasoulos T Leghi GE . Zinc is a critical regulator of placental morphogenesis and maternal hemodynamics during pregnancy in mice. Sci Rep. (2017) 7:15137. 10.1038/s41598-017-15085-2 29123159 PMC5680205

[80] Sun JY Jing MY Weng XY Fu LJ Xu ZR Zi NT . Effects of dietary zinc levels on the activities of enzymes, weights of organs, and the concentrations of zinc and copper in growing rats. Biol Trace Elem Res. (2005) 107:153–65. 10.1385/BTER:107:2:153 16217140

[81] Chen PH Wu J Xu Y Ding CC Mestre AA Lin CC . Zinc transporter zip7 is a novel determinant of ferroptosis. Cell Death Dis. (2021) 12:198. 10.1038/s41419-021-03482-5 33608508 PMC7895949

[82] Dixon SJ Lemberg KM Lamprecht MR Skouta R Zaitsev EM Gleason CE . Ferroptosis: an iron-dependent form of nonapoptotic cell death. Cell. (2012) 149:1060–72. 10.1016/j.cell.2012.03.042 22632970 PMC3367386

[83] Oh C Keats EC Bhutta ZA. Vitamin and mineral supplementation during pregnancy on maternal, birth, child health and development outcomes in low- and middle-income countries: a systematic review and meta-analysis. Nutrients. (2020) 12:491–20. 10.3390/nu12020491 32075071 PMC7071347

[84] Hamdan HZ Hamdan SZ Adam I. Association of selenium levels with preeclampsia: a systematic review and meta-analysis. Biol Trace Elem Res. (2022). 10.1007/s12011-022-03316-1 [Epub ahead of print]. 35687295

[85] Mardali F Fatahi S Alinaghizadeh M Kord Varkaneh H Sohouli MH Shidfar F . Association between abnormal maternal serum levels of vitamin B12 and preeclampsia: a systematic review and meta-analysis. Nutr Rev. (2021) 79:518–28. 10.1093/nutrit/nuaa096 33001182

[86] Yu Y Sun X Wang X Feng X. The association between the risk of hypertensive disorders of pregnancy and folic acid: a systematic review and meta-analysis. J Pharm Pharm Sci. (2021) 24:174–90. 10.18433/jpps31500 33878280

