# Supplementary material for: Maternal serum zinc level is associated with risk of preeclampsia: A systematic review and meta-analysis
Source: Front Public Health. 2022 Aug 1;10:968045. doi: 10.3389/fpubh.2022.968045 (PMC9376590; doi:10.3389/fpubh.2022.968045)
Supplement: Supplementary file 1 [file Data_Sheet_1.DOCX]

**PubMed**

#1 (‘’Zinc’’[Mesh]) OR (zinc[title/abstract]) OR (Zn[title/abstract]) OR zinc OR Zn

#2 ("Hypertension, Pregnancy-Induced"[Mesh] or (pregnancy (toxemia or toxaemia)) or preeclampsia or pre-eclampsia or eclampsia or HEELP syndrome or (Hemolysis, Elevated Liver enzymes and Low Platelets) or Pregnancy-Induced Hypertension or hypertension or pregnancy induced or gestational hypertension or transient hypertension)

#3 #1 AND #2

N=487

**Embase**

#1 'preeclampsia'/exp OR preeclampsia

#2 preeclampsia:ti,ab,kw OR 'hypertensive disorder of pregnancy':ti,ab,kw OR 'gestational hypertension':ti,ab,kw OR 'gestational hypertensive disorder':ti,ab,kw OR 'hypertensive disorder during pregnancy':ti,ab,kw OR 'pregnancy induced hypertension':ti,ab,kw OR 'pre-eclamptic toxaemia':ti,ab,kw OR ‘preeclamptic toxemia’:ti,ab,kw

#3 'zinc'/exp OR zinc OR zinc:ti,ab,kw OR Zn:ti,ab,kw

#4 #1 OR #2

#5 #3 AND #4

N=489

**Web of Science**

#1 TS=(Preeclampsia OR Eclampsia OR HELLP OR pre-eclampsia OR hypertensive disorder of pregnancy OR hypertensive disorder complicating pregnancy OR hypertensive disorder during pregnancy OR gestational hypertensive disorder OR HDCP OR gestational hypertension OR pregnancy induced hypertension OR preeclamptic toxemia OR preeclamptic toxaemia OR pre-eclamptic toxemia OR pre-eclamptic toxaemia)

#2 TS=(zinc OR Zn)

#3 #1 AND #2

N=295

**ClinicalTrials.gov**

Condition or disease: Pre-Eclampsia

Other terms: Zn or zinc

N=1

**African Journals Online (AJOL)**

‘zinc pre-eclampsia’

N=77

**Chinese electronic database:**

Wanfang, Chinese National Knowledge Infrastructure (CNKI) were applied with similar search strategy, using Chinese equivalent words of ‘Zinc and (pre-eclampsia or eclampsia or hypertensive disorder complicating pregnancy or HELLP syndrome)’ as two key words during search process.

N=49+9
