# Supplementary material for: Maternal serum zinc level is associated with risk of preeclampsia: A systematic review and meta-analysis
Source: Front Public Health. 2022 Aug 1;10:968045. doi: 10.3389/fpubh.2022.968045 (PMC9376590; doi:10.3389/fpubh.2022.968045)
Supplement: Supplementary file 2 [file Data_Sheet_2.docx]

**Supplementary Table 1. Characteristics of included studies**

| Included studies  (Author + year) | Country | Study type | Age of preeclampsia | | Age of control | | Zinc in PE (Mean±SD) | Zinc in control (Mean±SD) | Unit | Measure method |
| --- | --- | --- | --- | --- | --- | --- | --- | --- | --- | --- |
|  |  |  | N | (Mean±SD) | N | (Mean±SD) |  |  |  |  |
| Adam  2001[1] | Turkey | CC | 20 | 29±8 | 20 | 27±6.8 | 31.3±4.7 | 34.1±4.4 | ug/dL | FAAS |
| Ahsan  2013[2] | Bangladesh | CS | 44 | 26.05±5.41 | 27 | 24.11±4.93 | 0.016±0.002 | 0.015±0.002 | mmol/L | AAS |
| Akhtar  2011[3] | Bangladesh | CS | 60 | 25.11±5.66 | 30 | 25.20±4.85 | 902.5±157.15 | 1153.33±67.09 | ugm/L | FAAS |
| Akinloye 2010[4] | South Africa | CS | 49 | NG | 40 | NG | 8.6±1.4 | 9.4±0.8 | umol/L | FAAS |
| Al-Jameil 2014[5] | Saudi Arabia | CC | 40 | 31.55±6.14 | 40 | 31.20±5.84 | 0.67±0.59 | 1.30±0.83 | mg/L | ICP-OES |
| Al-Sakarneh  2021[6] | Jordan | CC | 30 | 28.3±0.8 | 30 | 28.0±0.9 | 63.71±1.24 | 65.37±1.27 | ug/dL | AAS |
| Al-Shalah 2015[7] | Iraq | CC | 60 | 27.72±0.66* | 60 | 26.85±0.53* | 57.28±1.74* | 87.54±3.71* | ug/dL | FGAAS |
| Atamer  2005[8] | Turkey | CS | 32 | 27.00±3.89 | 28 | 25.85±3.36 | 0.79±0.18 | 1.09±0.20 | mg/L | AAS |
| Bai  2013[9] | China | CC | 41+36 | 28.3±7.6 | 56 | 27.6±7.2 | 7.51±0.56/  6.39±0.33 | 8.96±0.63 | umol/L | NG |
| Bakacak 2015[10] | Turkey | CC | 38 | 29.2±3.56 | 40 | 28.7±3.82 | 81.24±12.06 | 108.45±0.63 | ug/dL | FAAS |
| Borella  1990[11] | Italy | CC | 24 | NG | 35 | NG | 10.49±2.28 | 9.60±2.29 | umol/L | AAS |
| Brito  2013[12] | Brazil | CC | 20+24 | 27.00±6.59 | 50 | 24.13±6.43 | 50.0±9.4/  38.8±8.2 | 48.3±8.3 | ug/dL | FAAS |
| Chababa  2016[13] | Zambia | CS | 41 | NG | 57 | NG | 89.17±47.19 | 76.20±35.23 | mg/dL | AAS |
| Desouky  2020[14] | Egypt | CC | 25 | 22.6±6.7 | 25 | 26.2±5.0 | 60.8±12.8 | 95.7±10.4 | mg/dL | NG |
| El-Moselhy  2010[15] | Egypt | CC | 100 | NG | 100 | NG | 60.81±9.74 | 95.70±12.41 | ug/dL | AAS |
| Elmugabil 2016[16] | Sudan | CC | 50 | 28.6±6.4 | 50 | 28.6±6.6 | 108.0±29.7 | 102.0±30.5 | ug/dL | AAS |
| Enebe  2020[17] | Nigeria | CS | 24+57 | 29.53±5.38 | 81 | 29.31±5.22 | 0.30±0.18/  0.45±0.44 | 0.54±0.80 | mg/L | AAS |
| Farzin  2012[18] | Iran | CC | 60 | 27.43±3.91 | 60 | 26.66±3.72 | 76.49±17.62 | 100.61±20.12 | ug/dL | FAAS |
| Feng  2013[19] | China | CC | 30 | 28.63±6.38 | 30 | 28.73±5.28 | 0.86±0.23 | 0.98±0.21 | ug/mL | AAS |
| Fenzl  2013[20] | Croatia | PC | 30 | 31.2 | 37 | 30.8 | 9.23±1.43 | 8.85±1.43 | umol/L | FAAS |
| Gan  2019[21] | China | CC | 33 | NG | 23 | NG | 10.30±2.98 | 13.44±2.33 | umol/L | AAS |
| Gao  2020[22] | China | CC | 427 | NG | 427 | NG | 6.33±1.75 | 6.49±1.84 | mg/L | ICP-MS |
| Gul  2022[23] | Turkey | CC | 43 | 30.2±6.8 | 45 | 27.2±5.3 | 75.1±20.9 | 80.3±17.7 | ug/dL | Fully automatic photometric method |
| Guo  2013[24] | China | CC | 26+20 | NG | 40 | NG | 61.06±6.24/  56.09±5.86 | 68.77±9.33 | umol/L | FAAS |
| Gupta  2014[25] | India | CC | 47+18 | NG | 75 | NG | 10.46±2.05/  9.28±1.63 | 10.63±1.82 | umol/L | AAS |
| Harma  2005[26] | Turkey | CC | 24 | 26.7±2.6 | 44 | 27.2±2.7 | 15.53±4.92 | 11.93±3.11 | ug/g | AAS |
| Hassan  2014[27] | Sudan | CC | 122 | 29.6±8.0 | 79 | 25.8±6.1 | 49.4±17.0 | 90.3±16.8 | ug/dL | AAS |
| Ikaraoha  2016[28] | Nigeria | CC | 59 | NG | 150 | NG | 45.8±9.7 | 68.2±10.1 | ug/dL | AAS |
| Illhan  2002[29] | Turkey | CC | 21 | NG | 30 | NG | 82.94±28.93 | 125.19±24.23 | ug/dL | AAS |
| Jain  2010[30] | India | CC | 25+25 | 23.04±3.76/  22.96±3.81 | 50 | 23.92±3.42 | 12.72±1.7/  12.04±1.4 | 15.64±2.40 | umol/L | AAS |
| Jamal  2017[31] | Pakistan | CS | 40 | 25.76±0.73 | 40 | 25.46±0.85 | 2.94±0.45 | 5.11±0.21 | mg/dL | FAAS |
| Ji  2010[32] | China | CC | 17+20 | 29.19 | 80 | 28.92 | 68.45±13.8/  66.55±13.5 | 66.45±12.45 | mmol/L | NG |
| Kanagal 2014[33] | India | CC | 60 | 27.45±4.33 | 60 | 25.87±3.11 | 8.84±0.87 | 14.87±0.89 | umol/L | AAS |
| Keshavarz 2017[34] | Iran | CC | 100 | 28.83±5.94 | 100 | 28.36±3.26 | 0.69±0.21 | 0.87±0.30 | mg/L | FAAS |
| Kolusari  2008[35] | Turkey | CC | 47 | 27.91±5.21 | 48 | 27.92±4.25 | 1.06±0.44 | 1.27±0.41 | ug/dL | AAS |
| Lewandowska 2019[36] | Poland | PC | 121 | 35.1±4.2 | 363 | 35.1±4.0 | 615.40±83.60 | 614.6±93.75 | ug/L | ICP-MS |
| Li  2009[37] | China | CC | 36+26 | 27.11±2.90 | 28 | 28.32±3.48 | 7.72±0.53/  6.10±1.40 | 8.91±0.60 | umol/L | Nitro-PAPS |
| Lu  2016[38] | China | CC | 60 | 29.55±2.20 | 30 | 29.60±2.00 | 6.94±1.13 | 8.93±0.54 | umol/L | AAS |
| Maduray 2017[39] | South Africa | CC | 43 | 25±5 | 23 | 24±5 | 18.03±30.28 | 2.13±3.01 | mg/L | ICP-OES |
| McKeating 2021[40] | Australia | NC | 44 | 31.55±3.8 | 193 | 32.24±3.91 | 526.93±111.59 | 510.15±175.70 | ug/L | ICP-MS |
| Memon  2017[41] | Pakistan | CC | 40 | NG | 40 | NG | 0.71±0.04 | 0.88±0.02 | mg/L | AAS |
| Mistry  2015[42] | UK / NZ /  Australia | NCC | 244 | 28 (23, 32)*** | 472 | 29 (23, 32)*** | 579.6 [521.1, 638.6]  *** | 575.7 [515.6, 641.7]  *** | ug/L | ICP-MS |
| Onyegbule 2016[43] | Nigeria | CC | 54 | 27±7.02 | 48 | 29±5.35 | 12.26±1.83 | 8.27±0.60 | umol/L | AAS |
| Pulei  2018[44] | Kenya | CC | 54 | NG | 54 | NG | 9.9±3.7 | 10.7±3.5 | umol/L | NG |
| Rafeeinia 2014[45] | Iran | CC | 50 | 26.50±3.90 | 50 | 27.10±4.60 | 0.71±0.26 | 0.73±0.33 | mg/L | AAS |
| Rathore 2011[46] | India | CC | 14 | NG | 47 | NG | 49.2±17.8 | 57.5±21.6 | ug/dL | AAS |
| Samar  2020[47] | China | CC | 50 | 29.34±4.35 | 52 | 26.44±4.02 | 85.43±12.07 | 91.79±9.02 | umol/L | Chemical light |
| Sarwar 2013[48] | Bangladesh | CC | 50 | 25.46±0.85* | 58 | 25.76±0.73* | 0.77±0.05* | 0.98±0.03* | mg/dL | FAAS |
| Ugwuja 2010[49] | Nigeria | CC | 40 | 29.45±3.70 | 40 | 27.55±4.23 | 9.97±9.74 | 10.87±10.28 | umol/L | AAS |
| Yang  2007[50] | China | CC | 47 | 28.75±2.64 | 30 | 26.83±2.59 | 0.76±0.14/  0.68±0.18 | 0.77±0.16 | ug/mL | FAAS |
| Yusrawati  2017[51] | Indonesia | CC | 70 | NG | 70 | NG | 4.80±2.62 | 5.50±3.53 | mg/L | NG |

NC: Nested cohort; NCC: Nested case-control; ICP-MS: Inductively coupled plasma mass spectrometry; ICP-OES: Inductively coupled plasma optical emission spectrometer; NG: Information not given in the article

*Mean ± SEM (standard error of mean); **Median range (Maximum-minimum); ***Median (Inter-quartile); #Median±SEM (Minimum, Maximum); ^+^A commercially available procedure for copper measurement

For those studies with sub-group of mild preeclampsia and severe preeclampsia, the mild usually comes first, followed with data of severe preeclampsia, and they were divided with the symbol ‘/’.

**Supplementary Table 2.1. Quality assessment (Modified NOS) for cross-sectional studies**

| Included studies | Selection | | | | Comparability | | Outcome | | Score |
| --- | --- | --- | --- | --- | --- | --- | --- | --- | --- |
|  | ① | ② | ③ | ④ | ⑤ | ⑥ | | ⑦ |  |
| Ahsan 2013[2] | ★ | ★ | ★ | ★★ | ★ | ★ | | ★ | 8 |
| Akhtar 2011[3] | ★ | ★ |  | ★ | ★ | ★ | | ★ | 6 |
| Akinloye 2010[4] | ★ |  |  | ★ | ★ | ★ | | ★ | 5 |
| Atamer 2005[8] | ★ | ★ | ★ | ★ | ★ | ★ | | ★ | 7 |
| Chababa 2016[13] | ★ | ★ |  | ★ | ★ | ★ | | ★ | 6 |
| Enebe 2020[17] | ★ | ★ | ★ | ★ | ★ | ★ | | ★ | 7 |
| Jamal 2017[31] |  | ★ | ★ | ★ |  | ★ | | ★ | 5 |

NOS: Newcastle-Ottawa Scale; NA: not applicable

Selection: ① Representativeness of the sample; ② Sample size; ③ Non-repondents; ④ Ascertainment of exposure (risk factors, maximum 2 stars);

Comparability (maximum 2 stars): ⑤ Comparability of outcomes based on the design or analysis;

Outcome (maximum 3 stars): ⑥ Assessment of outcome (maximum 2 stars); ⑦ Statistical test[52]

**Supplementary Table 2.2 Quality assessment (NOS) for cohort studies**

| Included studies | Selection | | | | Comparability | Outcome | | | Score |
| --- | --- | --- | --- | --- | --- | --- | --- | --- | --- |
|  | ① | ② | ③ | ④ | ⑤ | ⑥ | ⑦ | ⑧ |  |
| Fenzl 2013[20] | ★ | ★ | ★ | ★ | ★★ | ★ | ★ | ★ | 9 |
| Lewandowska 2019[36] | ★ | ★ | ★ | ★ | ★★ | ★ | ★ | ★ | 9 |
| McKeating 2021[40] | ★ | ★ | ★ | ★ | ★★ | ★ | ★ | ★ | 9 |

Selection: ① Representativeness of the exposed cohort; ② Selection of the non-exposed cohort; ③ Ascertainment of exposure; ④ Demonstration that outcome of interest was not present at the start of study;

Comparability: ⑤ Comparability of cohorts based on the design or analysis;

Outcome: ⑥ Assessment of outcome; ⑦ Was follow-up long enough for outcomes to occur; ⑧ Adequacy of follow up of cohorts

**Supplementary Table 2.3 Quality assessment (NOS) for case-control studies**

| Included studies | Selection | | | | | Comparability | | Exposure | | | | | | **Score** |
| --- | --- | --- | --- | --- | --- | --- | --- | --- | --- | --- | --- | --- | --- | --- |
|  | ① | ② | ③ | | ④ | ⑤ | ⑥ | | | | ⑦ | | ⑧ |  |
| Adam 2001[1] | ★ | ★ | ★ | |  | ★ | ★ | | ★ | | | | ★ | **7** |
| Al-Jameil 2014[5] | ★ |  |  | |  | ★★ | ★ | | ★ | | | | ★ | **6** |
| Al-Sakarneh 2021[6] | ★ | ★ | ★ | | ★ | ★★ | ★ | | ★ | | | | ★ | **9** |
| Al-Shalah 2015[7] | ★ | ★ | ★ | | ★ | ★★ | ★ | | ★ | | | | ★ | **9** |
| Bai 2013[9] | ★ | ★ | ★ | | ★ | ★★ | ★ | | ★ | | | | ★ | **9** |
| Bakacak 2015[10] | ★ |  | ★ | | ★ | ★★ | ★ | | ★ | | | | ★ | **8** |
| Borella 1990[11] | ★ |  |  | | ★ | ★★ | ★ | | ★ | | | | ★ | **7** |
| Brito 2013[12] | ★ | ★ | ★ | |  | ★ | ★ | | ★ | | | | ★ | **7** |
| Chababa 2018[13] | ★ |  | ★ | | ★ | ★★ | ★ | | ★ | | | | ★ | **8** |
| Desouky 2020[14] | ★ | ★ | ★ | |  | ★★ | ★ | | ★ | | | | ★ | **8** |
| El-Moselhy 2010[15] | ★ |  |  | |  | ★★ | ★ | | ★ | | | | ★ | **6** |
| Elmugabil 2016[16] | ★ |  |  | | ★ | ★★ | ★ | | ★ | | | | ★ | **7** |
| Farzin 2012[18] | ★ |  |  | | ★ | ★★ | ★ | | ★ | | | | ★ | **7** |
| Feng 2013[19] | ★ |  | ★ | | ★ | ★★ | ★ | | ★ | | | | ★ | **8** |
| Gan 2019[21] | ★ | ★ | ★ | | ★ | ★★ | ★ | | ★ | | | | ★ | **9** |
| Gao 2020[22] | ★ | ★ | ★ | |  | ★★ | ★ | | ★ | | | | ★ | **8** |
| Gul 2022[23] | ★ |  |  | | ★ | ★★ | ★ | | ★ | | | | ★ | **7** |
| Guo 2013[24] | ★ |  |  | | ★ | ★★ | ★ | | ★ | | | | ★ | **7** |
| Gupta 2014[25] | ★ |  |  | | ★ | ★★ | ★ | | ★ | | | | ★ | **7** |
| Hama 2005[26] | ★ |  |  | |  | ★★ | ★ | | ★ | | | | ★ | **6** |
| Hassan 2014[27] | ★ |  | ★ | | ★ | ★ | ★ | | ★ | | | | ★ | **7** |
| Ikaraoha 2016[28] | ★ |  | ★ | |  | ★ | ★ | | ★ | | | |  | **5** |
| Illhan 2002[29] | ★ |  | ★ | | ★ | ★★ | ★ | | ★ | | | | ★ | **8** |
| Jain 2010[30] | ★ |  | ★ | |  | ★★ | ★ | | ★ | | | | ★ | **7** |
| Ji 2010[32] | ★ | ★ | ★ | | ★ | ★★ | ★ | | ★ | | | | ★ | **9** |
| Kanagal 2014[33] | ★ |  |  | |  |  | ★ | | ★ | | | | ★ | **4** |
| Keshavarz 2017[34] | ★ | ★ |  | | ★ | ★ | ★ | | ★ | | | | ★ | **7** |
| Kolusari 2008[35] | ★ | ★ |  | |  | ★★ | ★ | | ★ | | | | ★ | **7** |
| Li 2009[37] | ★ |  |  | | ★ | ★★ | ★ | | ★ | | | | ★ | **7** |
| Lu 2016[38] | ★ |  | ★ | |  | ★★ | ★ | | ★ | | | | ★ | **7** |
| Maduray 2017[39] | ★ | ★ |  | |  | ★ | ★ | | ★ | | | | ★ | **6** |
| Memon 2017[41] | ★ |  | ★ | | ★ | ★★ | ★ | | ★ | | | | ★ | **8** |
| Mistry 2015[42] | ★ | ★ | ★ | | ★ | ★★ | ★ | | ★ | | | | ★ | **9** |
| Onyegbule 2016[43] | ★ |  | ★ | | ★ | ★ | ★ | | ★ | | | | ★ | **7** |
| Pulei 2018[44] | ★ | ★ |  | | ★ | ★ | ★ | | ★ | | | | ★ | **7** |
| Rafeeinia 2014[45] | ★ |  |  | |  | ★ | ★ | | ★ | | | | ★ | **5** |
| Rathore 2011[46] | ★ |  |  | |  |  | ★ | | ★ | | | | ★ | **4** |
| Samar 2020[47] |  |  |  | |  | ★★ | ★ | | ★ | | | | ★ | **5** |
| Sarwar 2013[48] | ★ | ★ | ★ | | ★ | ★ | ★ | | ★ | | | | ★ | **8** |
| Ugwuja 2010[49] | ★ |  | | ★ |  | ★ | | ★ | |  | | ★ | | **5** |
| Yang 2007[50] | ★ |  |  | | ★ | ★★ | | ★ | ★ | | | | ★ | **7** |
| Yusrawati 2017[51] | ★ | ★ | |  | ★ | ★ | | ★ | | ★ | | ★ | | **6** |

Selection: ① Is the case definition adequate; ② Representative of the cases; ③ Selection of controls; ④ Definition of controls

Comparability: ⑤ Comparability of cases and controls based on the design or analysis

Exposure: ⑥ Ascertainment of exposure; ⑦ Same method of ascertainment for cases and controls; ⑧ Non-response rate

**Supplementary Table 3 Sensitivity analysis of included studies**

| Study removed | Estimates after removing the study (95% CI) | I^2^ (%) |
| --- | --- | --- |
| Adam 2001[1] | -1.0064 [-1.3043, -0.7086] | 96.7% |
| Ahsan 2013[2] | -1.0285 [-1.3255, -0.7314] | 96.6% |
| Akhtar 2011[3] | -0.9812 [-1.2772, -0.6852] | 96.6% |
| Akinloye 2010[4] | -1.0057 [-1.3049, -0.7065] | 96.7% |
| Al-Jameil 2014[5] | -1.0017 [-1.3005, -0.7029] | 96.7% |
| Al-Sakarneh 2021[6] | -0.9926 [-1.2901, -0.6951] | 96.7% |
| Al-Shalah 2015[7] | -0.9918 [-1.2899, -0.6938] | 96.6% |
| Atamer 2005[8] | -0.9874 [-1.2844, -0.6905] | 96.7% |
| Bai 2013[9] | -0.9592 [-1.2492, -0.6692] | 96.5% |
| Bakacak 2015[10] | -0.9550 [-1.2467, -0.6632] | 96.5% |
| Borella 1990[11] | -1.0261 [-1.3234, -0.7288] | 96.7% |
| Brito 2013[12] | -1.0100 [-1.3095, -0.7105] | 96.7% |
| Chababa 2016[13] | -1.0256 [-1.3235, -0.7277] | 96.6% |
| Desouky 2020[14] | -0.9621 [-1.2561, -0.6682] | 96.6% |
| El-Moselhy 2010[15] | -0.9508 [-1.2348, -0.6668] | 96.3% |
| Elmugabil 2016[16] | -1.0234 [-1.3218, -0.7250] | 96.7% |
| Enebe 2020[17] | -1.0162 [-1.3173, -0.7151] | 96.7% |
| Farzin 2012[18] | -0.9934 [-1.2918, -0.6950] | 96.7% |
| Feng 2013[19] | -1.0082 [-1.3067, -0.7096] | 96.7% |
| Fenzl 2013[20] | -1.0240 [-1.3218, -0.7263] | 96.7% |
| Gan 2019[21] | -0.9961 [-1.2938, -0.6984] | 96.7% |
| Gao 2020[22] | -1.0227 [-1.3370, -0.7085] | 96.6% |
| Gul 2022[23] | -1.0141 [-1.3133, -0.7148] | 96.7% |
| Guo 2013[24] | -0.9941 [-1.2922, -0.6961] | 96.7% |
| Gupta 2014[25] | -1.0042 [-1.3049, -0.7035] | 96.7% |
| Harma 2005[26] | -1.0363 [-1.3318, -0.7408] | 96.6% |
| Hassan 2014[27] | -0.9671 [-1.2570, -0.6772] | 96.4% |
| Ikaraoha 2016[28] | -0.9713 [-1.2631, -0.6796] | 96.5% |
| Illhan 2002[29] | -0.9872 [-1.2840, -0.6903] | 96.7% |
| Jain 2010[30] | -0.9862 [-1.2831, -0.6894] | 96.6% |
| Jamal 2017[31] | -0.9111 [-1.1973, -0.6249] | 96.4% |
| Ji 2010[32] | -1.0210 [-1.3199, -0.7222] | 96.7% |
| Kanagal 2014[33] | -0.8932 [-1.1730, -0.6134] | 96.3% |
| Keshavarz 2017[34] | -1.0064 [-1.3086, -0.7043] | 96.7% |
| Kolusari 2008[35] | -1.0096 [-1.3092, -0.7101] | 96.7% |
| Lewandowska 2019[36] | -1.0218 [-1.3260, -0.7176] | 96.6% |
| Li 2009[37] | -0.9849 [-1.2815, -0.6883] | 96.6% |
| Lu 2016[38] | -0.9777 [-1.2730, -0.6823] | 96.6% |
| Maduray 2017[39] | -1.0310 [-1.3275, -0.7344] | 96.6% |
| McKeating 2021[40] | -1.0220 [-1.3215, -0.7225] | 96.6% |
| Memon 2017[41] | -0.9209 [-1.2081, -0.6337] | 96.4% |
| Mistry 2015[42] | -1.0235 [-1.3321, -0.7148] | 96.6% |
| Onyegbule 2016[43] | -1.0697 [-1.3535, -0.7858] | 96.3% |
| Pulei 2018[44] | -1.0153 [-1.3150, -0.7156] | 96.7% |
| Rafeeinia 2014[45] | -1.0182 [-1.3175, -0.7190] | 96.7% |
| Rathore 2011[46] | -1.0106 [-1.3086, -0.7126] | 96.7% |
| Samar 2020[47] | -1.0076 [-1.3073, -0.7079] | 96.7% |
| Sarwar 2013[48] | -1.0051 [-1.3048, -0.7054] | 96.7% |
| Ugwuja 2010[49] | -1.0175 [-1.3163, -0.7187] | 96.7% |
| Yang 2007[50] | -1.0120 [-1.3109, -0.7131] | 96.7% |
| Yusrawati 2017[51] | -1.0156 [-1.3161, -0.7150] | 96.7% |
| Pooled estimate | -0.9984 [-1.2923, -0.7045] | 96.6% |

**Reference**

1. Adam, B., et al., *Magnesium, zinc and iron levels in pre-eclampsia.* J Matern Fetal Med, 2001. **10**(4): p. 246-50.

2. Ahsan, T., et al., *Serum trace elements levels in preeclampsia and eclampsia: correlation with the pregnancy disorder.* Biol Trace Elem Res, 2013. **152**(3): p. 327-32.

3. Akhtar, S., S. Begum, and S. Ferdousi, *Calcium and zinc deficiency in preeclamptic women.* J Bangladesh Soc Physiol, 2011. **6**(2): p. 94-99.

4. Akinloye, O., O.J. Oyewale, and O.O. Oguntibeju, *Evaluation of trace elements in pregnant women with pre-eclampsia.* African Journal of Biotechnology, 2010. **9**(32): p. 5196-5202.

5. Al-Jameil, N., et al., *Analysis of serum trace elements-copper, manganese and zinc in preeclamptic pregnant women by inductively coupled plasma optical emission spectrometry: a prospective case controlled study in Riyadh, Saudi Arabia.* Int J Clin Exp Pathol, 2014. **7**(5): p. 1900-10.

6. Al-Sakarneh, N.A. and R.H. Mashal, *Evaluation of Zinc and Homocysteine Status in Pregnant Women and Their Association with Pre-eclampsia in Jordan.* Prev Nutr Food Sci, 2021. **26**(1): p. 21-29.

7. Al-Shalah, H.H., N.M. Al-Hilli, and M.A. Hasan, *The association of serum iron, zinc, and copper levels with preeclampsia.* Medical Journal of Babylon, 2015. **12**(4): p. 1027-1036.

8. Atamer, Y., et al., *Lipid peroxidation, antioxidant defense, status of trace metals and leptin levels in preeclampsia.* Eur J Obstet Gynecol Reprod Biol, 2005. **119**(1): p. 60-6.

9. Bai, T., *The diagnostic value of trace elements, LDH and UA in hypertensive disorders in pregnancy.* Chinese Journal of Postgraduate of Medicine, 2013. **36**(12): p. 49-52.

10. Bakacak, M., et al., *Changes in Copper, Zinc, and Malondialdehyde Levels and Superoxide Dismutase Activities in Pre-Eclamptic Pregnancies.* Med Sci Monit, 2015. **21**: p. 2414-20.

11. Borella, P., et al., *Maternal plasma concentrations of magnesium, calcium, zinc and copper in normal and pathological pregnancies.* Sci Total Environ, 1990. **99**(1-2): p. 67-76.

12. Brito, J.A., et al., *Enzyme activity of superoxide dismutase and zincemia in women with preeclampsia.* Nutr Hosp, 2013. **28**(2): p. 486-90.

13. Chababa, L., et al., *Relationship between Serum Zinc Levels and Preeclampsia at the University Teaching Hospital Lusaka, Zambia.* Medical Journal of Zambia, 2018. **43**: p. 5.

14. Elsayed El Desouky, et al., *Comparative Study for Serum Zinc and Copper Levels in Cases with Normal Pregnancy versus Preeclampsia.* Nature and Science, 2020.

15. El-Moselhy, E.A., H.H. Amin, and H.M.A. El-Aal, *Maternal serum calcium and trace elements; copper and zinc among preeclamptic women in Cairo, Egypt.* The Egyptian Journal of Hospital Medicine, 2010. **41**: p. 11.

16. Elmugabil, A., et al., *Serum Calcium, Magnesium, Zinc and Copper Levels in Sudanese Women with Preeclampsia.* PLoS One, 2016. **11**(12): p. e0167495.

17. Enebe, J.T., et al., *Serum antioxidant micronutrient levels in pre-eclamptic pregnant women in Enugu, south-East Nigeria: a comparative cross-sectional analytical study.* BMC Pregnancy Childbirth, 2020. **20**(1): p. 392.

18. Farzin, L. and F. Sajadi, *Comparison of serum trace element levels in patients with or without pre-eclampsia.* J Res Med Sci, 2012. **17**(10): p. 938-41.

19. Feng, J.J. and Y.X. Wang, *The measurement and clinical significance of serum trace elements in early - onset severe preeclampsia.* Chinese Journal of Birth Health & Heredity, 2013. **21**(05): p. 82-84.

20. Fenzl, V., et al., *Trace elements and oxidative stress in hypertensive disorders of pregnancy.* Arch Gynecol Obstet, 2013. **287**(1): p. 19-24.

21. Gan, Y., et al., *Correlation between Vitamin C, Vitamin E, trace element and preeclampsia during pregnancy.* Chin J Clin Obstet Gynecol, 2019. **20**(05): p. 456-457.

22. Gao, L.Y., et al., *Relationship between newborn birth weight and serum zinc of pregnant women with preeclampsia.* Chinese Remedies & Clinics, 2020.

23. Gul, A.Z., et al., *Maternal Serum Levels of Zinc, Copper, and Thiols in Preeclampsia Patients: a Case-Control Study.* Biol Trace Elem Res, 2022. **200**(2): p. 464-472.

24. Guo, L.L., et al., *The study on the relationship between trace elements content in whole blood and hypertensive disorder complicating pregnancy.* Chinese Journal of Birth Health & Heredity, 2013(11): p. 4.

25. Gupta, S., et al., *Plasma and erythrocyte zinc in pre-eclampsia and its correlation with foetal outcome.* J Assoc Physicians India, 2014. **62**(4): p. 306-10.

26. Harma, M., M. Harma, and A. Kocyigit, *Correlation between maternal plasma homocysteine and zinc levels in preeclamptic women.* Biol Trace Elem Res, 2005. **104**(2): p. 97-105.

27. Hassan, E.E., et al., *Assessment of trace elements in Sudanese preeclamptic pregnant women.* European Journal of Biomedical and Pharmaceutical Sciences, 2014. **1**(2): p. 8.

28. Ikaraoha, I.C., et al., *Serum trace metals in Pre-Eclamptic Nigerians.* Asian Journal of Medical Sciences, 2016. **7**(3): p. 78-83.

29. Ilhan, N., N. Ilhan, and M. Simsek, *The changes of trace elements, malondialdehyde levels and superoxide dismutase activities in pregnancy with or without preeclampsia.* Clin Biochem, 2002. **35**(5): p. 393-7.

30. Jain, S., et al., *The role of calcium, magnesium, and zinc in pre-eclampsia.* Biol Trace Elem Res, 2010. **133**(2): p. 162-70.

31. Jamal, B., F. Shaikh, and M.Y. Memon, *To Determine the Effects of Copper, Zinc and Magnesium in Patients with Pre-Eclampsia.* Journal of the Liaquat University of Medical and Health Sciences, 2017. **16**(1): p. 53-57.

32. Ji, C., et al., *The Clinical Implication of Serum Trace Elements in Patients with Hypertensive Disorder in Pregnancy.* Medical Information, 2010. **23**(11).

33. Kanagal, D.V., et al., *Zinc and copper levels in preeclampsia: a study from coastal South India.* 2014. **3**(2): p. 370-373.

34. Keshavarz, P., et al., *Alterations in Lipid Profile, Zinc and Copper Levels and Superoxide Dismutase Activities in Normal Pregnancy and Preeclampsia.* Am J Med Sci, 2017. **353**(6): p. 552-558.

35. Kolusari, A., et al., *Catalase activity, serum trace element and heavy metal concentrations, and vitamin A, D and E levels in pre-eclampsia.* J Int Med Res, 2008. **36**(6): p. 1335-41.

36. Lewandowska, M., et al., *First Trimester Serum Copper or Zinc Levels, and Risk of Pregnancy-Induced Hypertension.* Nutrients, 2019. **11**(10).

37. Li, P.Z. and X.Y. Li, *Trace Elements in Pregnancy-Induced Hypertension and Related Diseases Research.* Guide of China Medicine, 2009(10): p. 29-31.

38. Lu, Y.H., et al., *Correlation between hypertensive disorder in pregnancy and serum calcium, prostaglandin E, endothelin.* Hebei Medical Journal, 2016. **38**(07): p. 1057-1059.

39. Maduray, K., et al., *Elemental analysis of serum and hair from pre-eclamptic South African women.* J Trace Elem Med Biol, 2017. **43**: p. 180-186.

40. McKeating, D.R., et al., *Circulating trace elements for the prediction of preeclampsia and small for gestational age babies.* Metabolomics, 2021. **17**(10): p. 90.

41. Memon, A.R., et al., *Association of Serum Zinc level with Pre Eclampsia.* Journal of the Liaquat University of Medical and Health Sciences, 2017. **16**(1): p. 58-61.

42. Mistry, H.D., et al., *Association between maternal micronutrient status, oxidative stress, and common genetic variants in antioxidant enzymes at 15 weeks׳ gestation in nulliparous women who subsequently develop preeclampsia.* Free Radic Biol Med, 2015. **78**: p. 147-55.

43. Onyegbule, A.O., et al., *Serum copper and zinc levels in preeclamptic Nigerian women.* Niger Med J, 2016. **57**(3): p. 182-4.

44. Pulei AN, Kinuthia J, and O. Omondi, *Serum levels of selected micronutrients in primigravida with pre-eclampsia versus their normotensive counterparts.* EPH - International Journal of Medical and Health Science, 2018.

45. Rafeeinia, A., et al., *Serum copper, zinc and lipid peroxidation in pregnant women with preeclampsia in gorgan.* Open Biochem J, 2014. **8**: p. 83-8.

46. Rathore, S., et al., *Comparative study of trace elements and serum ceruloplasmin level in normal and pre-eclamptic pregnancies with their cord blood.* Biomedical Research-India, 2011. **22**(2): p. 207-210.

47. Samar, A., D.L. Wang, and X. Iskandar, *Correlation between gestational hypertension and serum vitamin D, trace element level* Journal of Xinjiang Medical University, 2020. **43**(5): p. 597-600,606.

48. Sarwar, M.S., et al., *Comparative study of serum zinc, copper, manganese, and iron in preeclamptic pregnant women.* Biol Trace Elem Res, 2013. **154**(1): p. 14-20.

49. Ugwuja, E.I., et al., *Comparison of plasma copper, iron and zinc levels in hypertensive and non-hypertensive pregnant women in Abakaliki, South Eastern Nigeria.* Pak J Nutr, 2010. **9**(12): p. 1136-40.

50. Yang, L.T., et al., *The study on the relationship between hypertensive disorder complicating pregnancy and the serum zinc, copper, iron, maganese.* Maternal and Child Health Care of China, 2007(29): p. 4082-4085.

51. Yusrawati, et al., *Analyses of Nutrients and Body Mass Index as Risk Factor for Preeclampsia.* J Obstet Gynaecol India, 2017. **67**(6): p. 409-413.

52. Modesti, P.A., et al., *Panethnic Differences in Blood Pressure in Europe: A Systematic Review and Meta-Analysis.* PLoS One, 2016. **11**(1): p. e0147601.
