# Supplementary material for: Maternal serum zinc level is associated with risk of preeclampsia: A systematic review and meta-analysis
Source: Front Public Health. 2022 Aug 1;10:968045. doi: 10.3389/fpubh.2022.968045 (PMC9376590; doi:10.3389/fpubh.2022.968045)
Supplement: Supplementary file 3 [file Data_Sheet_3.DOCX]

|  |
| --- |
| **(a)** |
|  |
| **(b)** |

**Supplementary Figure 1.** Assessment of publication bias. **(a).** Begg’s test; **(b).** Egger's test

**Supplementary Figure 2.** Sensitivity analysis of included studies. Detailed data can be seen in Supplementary Table 3.

|  |
| --- |
| **(a)** |
| **** |
| **(b)** |
| **** |
| **(c)** |

**Supplementary Figure 3.** Meta-regression of the included studies: **(a).** the geographical location of the study (Others stand for studies from Australia, Brazil, Croatia, New Zealand, Poland, and the UK); **(b).** the measuring methods of serum Zn (AAS/FAAS, ICP-MS, other methods); **(c).** the study types.


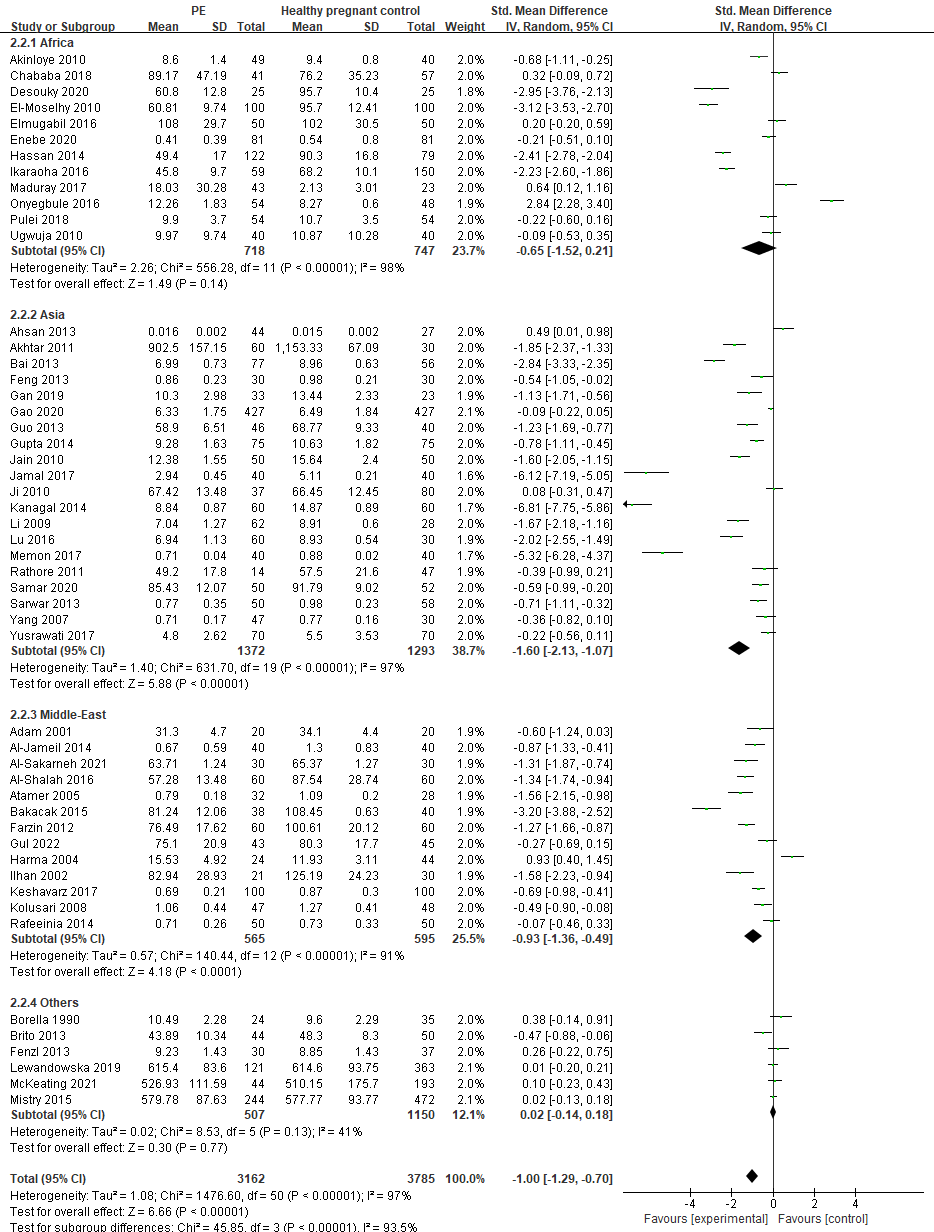


**Supplementary Figure 4**. Maternal serum zinc levels in preeclamptic and healthy pregnant women from Africa, Asia, Middle-East and other regions of the world (Italy, Brazil, Croatia, Poland, Australia, UK, New Zealand)

**
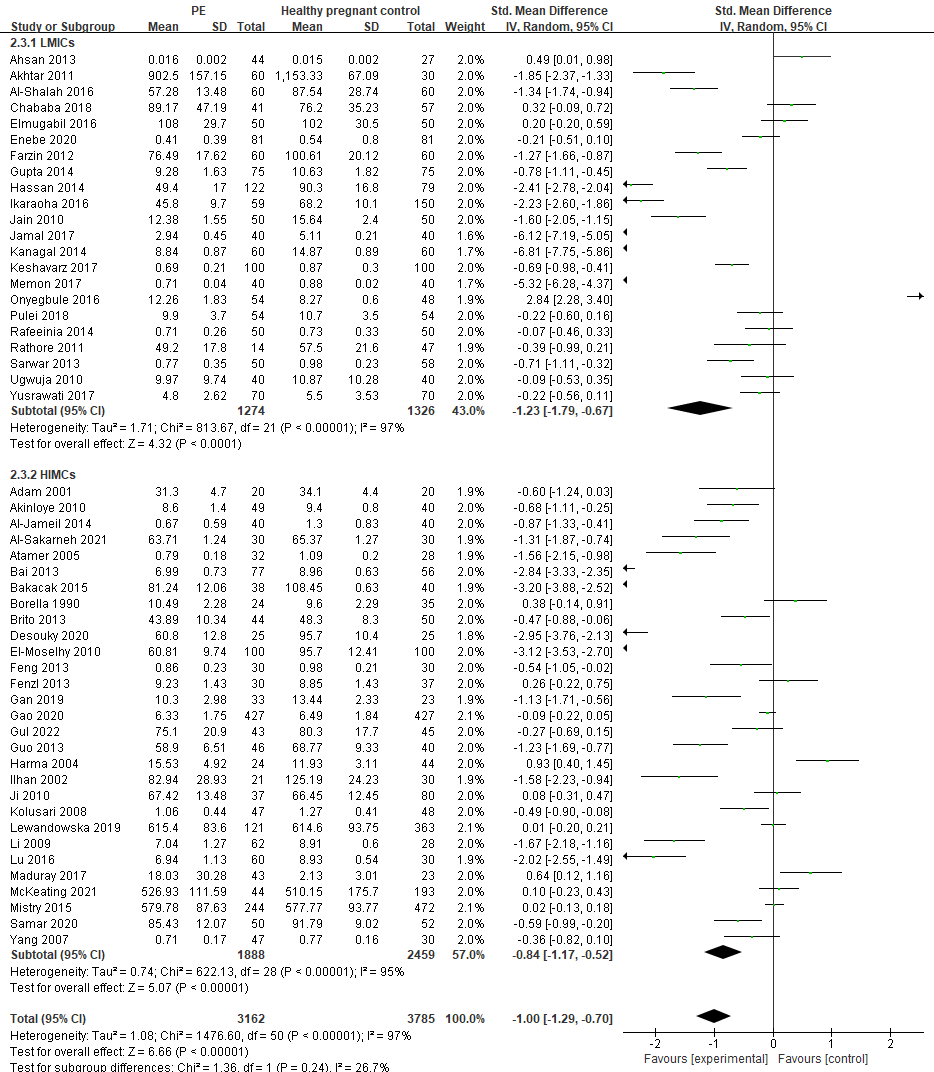
**

**Supplementary Figure 5.** Maternal serum zinc levels in preeclamptic and healthy pregnant women from LMICs Vs HMICs


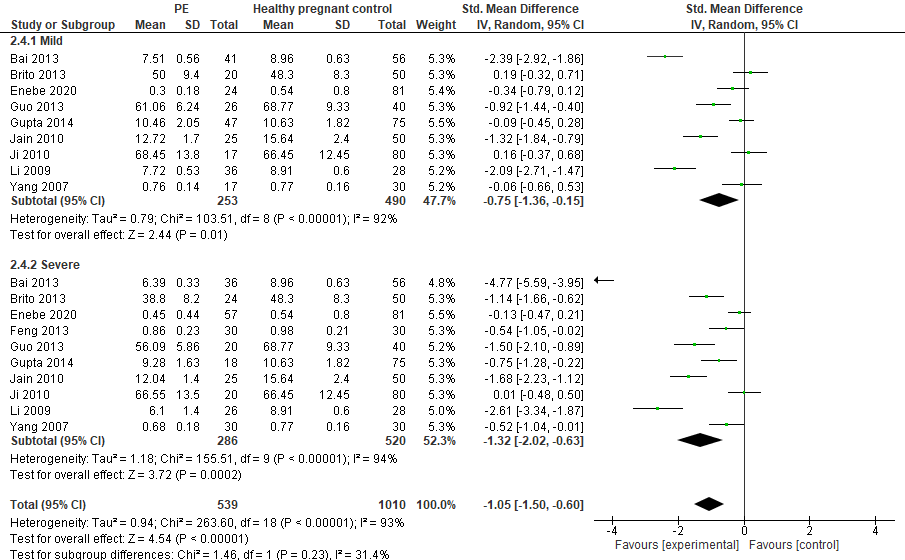


**Supplementary Figure 6.** Maternal serum zinc levels in women with mild Vs severe preeclampsia compared with control
